# Metagenomic Insight into Environmentally Challenged Methane-Fed Microbial Communities

**DOI:** 10.3390/microorganisms8101614

**Published:** 2020-10-20

**Authors:** Yue Zheng, Huan Wang, Zheng Yu, Fauzi Haroon, Maria E. Hernández, Ludmila Chistoserdova

**Affiliations:** 1 CAS Key Laboratory of Urban Pollutant Conversion, Institute of Urban Environment, Chinese Academy of Sciences, Xiamen 361021, China; yzheng@iue.ac.cn (Y.Z.); huanwang@iue.ac.cn (H.W.); 2 Department of Chemical Engineering, University of Washington, Seattle, WA 98195, USA; yuzheng@csu.edu.cn; 3 Department of Organismic and Evolutionary Biology, Harvard University, Cambridge, MA 02138, USA; fauziharoon@gmail.com; 4 Biotechnological Management of Resources Network, Institute of Ecology A. C., 91070 Xalapa, Mexico

**Keywords:** methanotrophic communities, microcosm incubation, metagenomics, metatranscriptomics, *Methylococcaceae*, *Methylophilaceae*, Burkholderiales, Bacteroidetes, *Lacunisphaera*, *Bacteriovorax*

## Abstract

In this study, we aimed to investigate, through high-resolution metagenomics and metatranscriptomics, the composition and the trajectories of microbial communities originating from a natural sample, fed exclusively with methane, over 14 weeks of laboratory incubation. This study builds on our prior data, suggesting that multiple functional guilds feed on methane, likely through guild-to-guild carbon transfer, and potentially through intraguild and intraspecies interactions. We observed that, under two simulated dioxygen partial pressures—low versus high—community trajectories were different, with considerable variability among the replicates. In all microcosms, four major functional guilds were prominently present, representing *Methylococcaceae* (the true methanotrophs), *Methylophilaceae* (the nonmethanotrophic methylotrophs), Burkholderiales, and Bacteroidetes. Additional functional guilds were detected in multiple samples, such as members of Opitutae, as well as the predatory species, suggesting additional complexity for methane-oxidizing communities. Metatranscriptomic analysis suggested simultaneous expression of the two alternative types of methanol dehydrogenases in both *Methylococcaceae* and *Methylophilaceae*, while high expression of the oxidative/nitrosative stress response genes suggested competition for dioxygen among the community members. The transcriptomic analysis further suggested that Burkholderiales likely feed on acetate that is produced by *Methylococcaceae* under hypoxic conditions, while Bacteroidetes likely feed on biopolymers produced by both *Methylococcaceae* and *Methylophilaceae*.

## 1. Introduction

Aerobic methanotrophs are a group of organisms that play an important role in mitigating methane emissions from both natural (wetlands, lakes, and soils) and anthropogenic (rice paddies, landfills, oil, gas, and coal mining) sources [1,2,3]. Over the past decades, these organisms have been studied as pure cultures and under conditions of the excess of dioxygen [4,5]. However, more recently, a concept of communal metabolism of methane has become prominent, providing a more complex outlook at the environmental function of the aerobic methanotrophs [6,7,8]. Another conceptual change in understanding the aerobic methanotrophy involves the realization that aerobic methanotrophs are likely dioxygen-limited in their natural environments, and, in many studies, they have been inferred to be active under hypoxic and even anoxic conditions [9,10,11,12]. Furthermore, oxygen sensitivity has been demonstrated for some aerobic methanotrophs [13,14]. Thus, experimental designs involving high dioxygen partial pressures may not be optimal for addressing the metabolic features of these microbes and the communities they form. As to the nature of the community composition and function, so far, *Methylophilaceae* methylotrophs have been identified as some of the most persistent partners in methane oxidation by *Methylococcaceae* [15,16,17], and the major metabolite transferred from the methanotrophs to the *Methylophilaceae* appears to be methanol [18]. Moreover, interspecies interactions involving the switch between alternative methanol dehydrogenase enzymes have been inferred [18]. However, it still remains unclear whether the relationships between *Methylococcaceae* and *Methylophilaceae* are mutualistic, and whether the *Methylophilaceae* provide something to the methanotrophs or they simply scavenge the spilled methanol. Even less is known about other members of the communities and their potential interactions with the methanotrophs. The persistent presence of certain nonmethylotrophic taxa has been reported, such as Burkholderiales and Bacteroidetes [6,15,16,17], but it remains unknown which metabolites support their respective populations, how these specific taxa are selected, and whether there is a positive feedback between these populations and the methanotroph populations. Several recent proof-of-principle studies have demonstrated the utility of simple synthetic communities in addressing the mechanistic details of communal behavior in the methane utilization and carbon flow among the community members [18,19]. However, further insights are necessary to identify the optimal models for such experiments and to build collections of model organisms that reflect the composition of communities in natural habitats.

In this paper, we describe our efforts to obtain a high-resolution set of data on microbial communities persisting in laboratory microcosms fed with methane as a single carbon source, under two different dioxygen partial pressures, in order to elicit further information on the complexity of methane-oxidizing communities and on the identity of the most persistent partners in methane oxidation, even if these might be present at low abundances. To better reconstruct the metabolism of the lower abundance partners, specifically the Burkholderiales and the Bacteroidetes, we cultivated representatives of the relevant species and sequenced their genomes. Overall, we here present a comprehensive (meta)genomic/metatranscriptomic dataset for a manipulated lake sediment methanotroph community, over a timescale, along with a collection of model organisms that should be useful for employment in synthetic communities of varied complexity.

## 2. Materials and Methods

### 2.1. Experimental Design for Microcosm Manipulation

**Figure 1:**
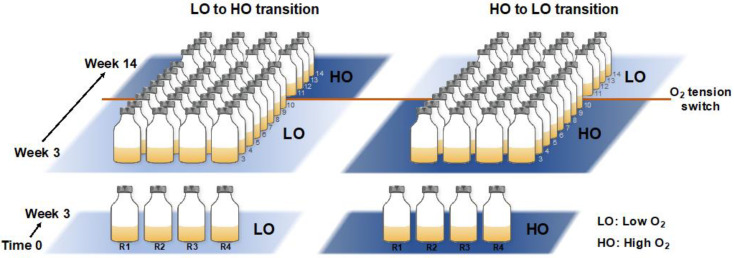
Schematic of the microcosm incubation design. Bottom panel: Four replicate cultures were established at two O_2_ tensions, low (LO) and high (HO). Top panel: After 3 initial weeks, weekly transfers were carried out along with DNA/RNA sampling from week 4 through week 14. At week 10, the conditions were switched from LO to HO and from HO to LO, respectively, to mimic environmental perturbance.

A total of eight microcosms were established by combining 5 mL of previously frozen sediment sample, supplemented with dimethyl sulfoxide (DMSO) for cell integrity preservation [15], with 100 mL of ½ strength nitrate minimal saline medium (NMS), in 250 mL vials sealed with rubber stoppers [15]. We previously established that the trajectories of the communities established with either fresh or frozen sediment are essentially superimposable, based on the 16S rRNA gene diversity analysis [15]. Thus, the integrity of the entire community was assumed to be preserved by this storage method. Half of the microcosms received 15% air/25% methane/60% dinitrogen, designated as ‘low-oxygen’ (LO) microcosms (calculated 45 µM dissolved O_2_ concentration; replicates LO1-LO4). Another half received 75% air/25% methane, designated as ‘high-oxygen’ (HO) microcosms (calculated 225 µM dissolved O_2_ concentration, replicates HO1-HO4). Methane (calculated dissolved concentration 1.5 mM) was used as a single carbon source to drive community metabolism. Vials were incubated at 18 °C with shaking (250 rpm). After 3 initial weeks of incubation, samples were transferred with dilutions, 10% of the original volume for the HO samples and 20% of the original volume for the LO samples, to maintain the optimal level of biomass, as determined empirically. At week 10, the conditions were perturbed by switching the LO samples to HO condition and, respectively, HO samples to LO condition, to mimic a natural environmental perturbance. Whereas it is common for laboratory experiments to employ contrasting conditions, in order simplify the system and to decrease the duration of the experiment, such perturbance events can be imagined in real life. For example, a movement of a warm or a larva through the sediment may lead to abrupt oxygenation, while an algal bloom above the sediment may lead to abrupt hypoxia. Samples for DNA/RNA extraction were taken starting with week 4, and repeated every week throughout the experiment, with a total of 11 samples for each replicate (88 total). The schematic for the experimental design is presented in Figure 1.

### 2.2. DNA and RNA Extraction, Sequencing and Analysis 

DNA and RNA were extracted following the published protocols, and their quantity and quality were monitored as previously described [12,15,16,17,18]. The preparations were mailed on dry ice to the DOE Joint Genome Institute (JGI) facility for analysis. The JGI pipelines of the time (circa 2015) were employed to sequence the DNA and the RNA samples, and genome assembly was originally performed in 2015 and subsequently updated in 2018 (the SPAdes assembly) [20]. Data from both assemblies, as well as data for the assembled metatranscriptomes, are available at the IMG/M site (https://img.jgi.doe.gov/) under the study name ‘Freshwater sediment methanotrophic microbial communities from Lake Washington under simulated oxygen tension’. Note that ELVIZ analysis, which is a convenient tool for visualizing relative abundances of community members at different phylogenetic classification levels [21], has been performed on all the metagenomic datasets. These are accessible via the public IMG/M interface.

Metaganome assembled genomes (MAGs) were constructed using the MetaBAT software [22], as part of the IMG/M pipeline [23].

Species abundances in each metagenome and metatranscriptome were determined by mapping reads to the assembled scaffolds (the SPAdes assembly) using Bowtie2 [24], and the taxonomic abundance (*TA*), at a desired taxonomic level, was normalized by the length of the scaffold as follows. The *TA* was summarized based on the taxonomic annotation of each scaffold.

(1) $$TA=\frac{rn\times rl\times 10^{6}}{sl\times T_{TA}}$$

where *rn* is the counts of reads mapped to the scaffold, *rl* is the read length, *sl* is the scaffolds length, and *T_TA_* is the sum of *rn·rl·sl*^−1^ for all scaffolds.

For principal component analysis (PCA), the R package was employed, based on a matrix of relative abundances of the core taxa (*Methylococcaceae, Methylophilaceae*, Burkholderiales, Bacteroidetes), in the eight samples, over 11 sampling points.

To address expression of specific genes (*xoxF*, *mxaF*, etc.), the select target genes were used, originating from single species genomes. To evaluate expression of *xoxF* versus *mxaF*, a database of genes has been compiled for *Methylophilaceae*, and the sequences were sorted into genotypes based on >5% divergence, at the protein level. A single representative of each genotype was used. In all other cases, a single proxy gene was used. The respective gene and protein IDs are listed in Table S1. Metatranscriptomic reads were aligned with these target genes using Bowtie2 [24]. The alignments were postprocessed into sorted BAM files with Samtools [25]. Reads were attributed to open reading frames (ORFs) using the htseq count tool from the HTseq [26]. TPM (transcripts per million) values were calculated as follows:

(2) $$TPM=\frac{rg\times rl\times 10^{6}}{cl\times T_{TPM}}$$

where *rg* is the reads mapped to the gene, *rl* is the read length, *cl* is the coding sequence (CDS) length, and *T_TPM_* is the sum of *rg·rl·cl*^−1^ for all genes.

To identify highly expressed genes in *Acidovorax* and *Flavobacterium*, reads from all 88 metatranscriptomes were originally matched to select proxy genomes, *Acidovorax radiicis* N35 (prior to availability of the native isolates; the genome is part of the IMG/M database) and *Flavobacterium* sp. 83 and *Flavobacterium* sp. Fl [27], respectively, using the JGI pipeline. Samples with the highest number of matches were selected, and the highly expressed genes were further employed to match transcripts to the MAGs and the isolate genomes of *Acidovorax* and *Flavobacterium*, as follows. Datasets of relevant *Acidovorax* (a total of 16) and *Flavobacterium* (a total of 20) MAGs and genomes were constructed, and reads from metatranscriptomes HO 10, R1 and/or HO 14, R3 were matched to these databases, as described above. The reads matching to all the respective gene homologs, as determined by BLAST, were then summed to reflect transcription of a consensus gene. Read counts were normalized per gene length and expressed as TPM, per total TPM classified as *Acidovorax* or *Flavobactertium*, respectively.

### 2.3. Isolation and Identification of Model Organisms

Representatives of Burkholderiales and Flavobacteriales were isolated essentially as previously described [19]. Briefly, frozen samples of Lake Washington sediment were used to establish microcosms, as described above, and were incubated under HO or LO conditions with transfers, as described [15,16]. Dilutions of the resulting cultures were plated onto R2A agar plates (Thermo Fisher Scientific, Waltham, MA, USA) or diluted R2A plates (1/2 and 1/5 dilutions), and plates were incubated at room temperature (approximately 24 °C) for up to 2 weeks, until clearly visible colonies appeared. The colonies were examined for shape and color, and representatives were purified through serial transfers onto the same medium. DNA was then isolated, and 16S rRNA genes were sequenced as previously described [17]. Cultures that appeared most distinct in their phenotypes/genotypes were chosen for genome sequencing. Genome sequencing was carried out by the JGI facility, and genomic data are available through the IMG/M website (https://img.jgi.doe.gov/). The list of the genomes generated in this study is presented in Table S2. For comparisons between the model organism genomes and the MAGs generated in this study, average nucleotide identity (ANI) estimates were carried out using pyani.py (https://github.com/widdowquinn/pyani), employing BLASTN to align the genomic fragments.

## 3. Results

### 3.1. Community Dynamics are Dominated by Four Major Phyla, Firmly Defining the ‘Core Microbiome’ Involved in Methane Consumption

**Figure 2:**
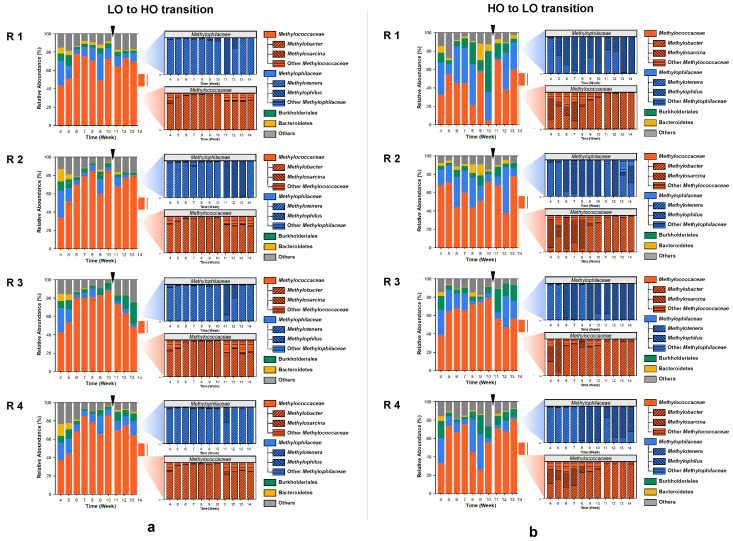
Community dynamics in microcosms transitioning from ‘low’ to ‘high’ (**a**) and from ‘high’ to ‘low’ (**b**) oxygen conditions, at the family/order level (left) and at the genus level (right). The switch between the conditions at week 10 is marked by an arrow. R1–R4, replicates.

A total of 88 metagenomes and respective metatranscriptomes representing four-time replicated time series, were sequenced producing metagenomes of 13,054,964 to 164,133,484 base pairs in size, encoding 18,370 to 324,524 genes (the SPAdes assembly) and metatranscriptomes of 5,358,049 to 60,829,165 base pairs in size (assembled sequences) representing 16,104 to 165,922 genes. Community composition for each metagenome and metatranscriptome was calculated using the IMG/M pipelines [20]. These analyses uncovered the preeminent presence, at the phylum level, of Proteobacteria, with a minor but persistent presence of Bacteroidetes (Figure 2). At the order/family level, community dynamics were dominated by the Methylococcales/*Methylococcceae* and Nitrosomonadeles/*Methylophilaceae* (Figure 2), as expected based on prior studies that employed 16S rRNA-based methods as well as (shallow) metagenomics [15,16]. In addition, members of Burkholderiales and Bacteroidetes were prominently and persistently present in each microcosm. Although minor in most microcosms, the populations of Burkholderiales were recorded at as high as >40% of total reads (HO1, week 10), and Bacteroidetes were recorded at as high as 20% of total reads (HO1, week 9). Overall, the dynamics observed among the four replicates, under each of the experimental conditions, indicated high stochasticity, with the populations of the major taxa shifting significantly over time. Among the replicates, the ‘high’ dioxygen condition microcosms showed the most of variation, in accord with the previously published data [15,16]. For example, HO1 appeared to have a community ‘crash’ at week 10, where the population of *Methylococcales* decreased to less than 5%, while the population of *Burkholderiales* soared to over 40%. PCA analysis employing abundances of these core taxa showed significant separation between the LO and the HO microcosms (Figure S1), in agreement with prior analyses based on the 16S rRNA gene diversity, applied to similarly run microcosms [15]. 

Transcript distribution among the four major taxa essentially followed the pattern observed with the DNA read distribution (Figure S2). As expected, the communal transcriptomes were dominated by the *Methylococcaceae* transcripts, due to their high relative abundance in most microcosms, as well as the inherently high expression of the methane monooxygenase genes [17].

### 3.2. Community Dynamics within Methylococcaceae and Methylophilaceae Reveal Differential Response to Dioxygen Supply

Under the ‘low’ oxygen (LO) condition, the *Methylococcaceae* community was overwhelmingly dominated by *Methylobacter* species, with the exception of the very early samples and the post-disturbance samples that contained other gammaproteobacterial methanotrophs (Figure 2a). The *Methylophilaceae* present in these communities were mostly *Methylotenera* species, with the exception of the early post-disturbance samples in which significant (up to nearly 100%) shifts toward *Methylophilus* species occurred in most microcosms, albeit temporarily (Figure 2a). The dynamics were quite different in the communities supplied with the ‘high’ concentration of dioxygen (HO), selecting for *Methylosarcina* and other *Methylococcaceae*, in addition to *Methylobacter*, especially in the early samples (Figure 2b). After the switch to the LO condition, the *Methylobacter* species rapidly outcompeted all other methanotroph genera (Figure 2b). The dynamics among the *Methylophilaceae* were also quite different compared to the LO condition, where the *Methylophilus* species gradually outcompeted the *Methylotenera* species, and even after the perturbance, *Methylotenera* only surged temporarily, being quickly replaced by *Methylophilus* (Figure 2a). Overall, the dynamics observed in this study supported prior observations based on 16S rRNA analyses, highlighting the differential roles among the species of *Methylococcaceae* and *Methylophilaceae* in response to dioxygen partial pressures and further suggesting that, while similar in their core metabolisms [28,29,30], specific members of *Methylococcaceae* and *Methylophilaceae* tend to select for specific environmental niches, with one of the major factors being the dioxygen partial pressure [15,16].

### 3.3. Metagenome-Based Genome Assemblies Provide Proxies for Major Players in the Communal Methane Consumption

We carried out the initial MAG assembly efforts early after the metagenomic data were available, circa 2015, and these resulted in a handful of ‘high’-quality MAGs, based on the standards of the time. These efforts resulted in recovery of MAGs representing the genomes of *Methylococcaceae, Methylophilaceae*, Burkholderiales, and Bacteroidetes, as expected (Table S3). Less expectedly, high-quality bins were also produced representing Opitutae and Bdellovibrionales (Table S3). We later carried out MAG assembly using the current JGI MAG binning pipeline [23]. Of these, 329 qualified as high- or medium-quality MAGs, with each MAG represented by 1 to 996 scaffolds (Table S4). Of the MAGs, 90 were classified as *Methylophilaceae*, 63 as *Methylococcaceae*, 63 as Burkholderiales, and 53 as Bacteroidetes, supporting prior data on the persistence of these organisms in methane-consuming communities [15,16]. MAGs were also assembled that represented Bdellovibrionales/ *Bacteriovoracaceae*, the predatory species known to feast on proteobacterial hosts [31], as well as another predatory type belonging to Polyangiales. Additional MAGs represented Opitutae classified as *Lacunisphaera* (a total of 9) and Proteobacteria of the orders Xanthomonadales (*Thermomonas fusca*, a total of 12), Rhodocyclales (*Sulfuritalea* and *Dechloromonas*, a total of 7), Pseudomonadales (*Pseudomonas*, a total of 4), and Thiotrichales (a total of 2). Whereas these latter types were not persistently present in every microcosm and throughout the duration of the experiment, they do represent additional players that may be involved in the dynamics of the methanotroph communities.

Genome-genome comparisons and metabolic reconstruction determined that the Xanthomonadales, Rhodocyclales, and Pseudomonadales MAGs represented species closely related to previously described species, with their relatively large (>5 Mb) genomes encoding a variety of functions for carbon and nitrogen metabolisms (at least partial denitrification). None of them encoded the methylotrophy functions (not shown). MAGs belonging to other taxa represented novel species (see below).

### 3.4. Organisms Representing the Core Microbiota, Burkholderiales and Bacteropidetes Isolated from Methane-Consuming Microcosms Provide High-Quality Genomic Scaffolds

MAGs, while providing vital genomic information for uncultivated organisms, are still only suggestive of the genomic content of the respective organisms, and most of them could not be considered as ‘complete’ genomes. Thus, we made an effort to isolate some major species detected as core microbiota in the methane-consuming microcosms manipulated with the same sediment sample, collected in 2013 [15]. After some trial-and-error attempts, such as enriching with acetate (for *Acidovorax*), we settled on a protocol of enriching under standard conditions (‘low’ or ‘high’ dioxygen), as described, and plating onto R2A agar or dilute R2A agar. By doing so, we were able to isolate numerous species that represented the desired functional guilds, Burkholderiales and Bacteroidetes. We sequenced, respectively, 25 and 13 genomes representing these functional guilds (Table S2). Whereas attempts were made to isolate *Bacteriovoracaceae* and Polyangiales, these were not successful. Note that the species belonging to *Methylococcaceae*, *Methylophilacaea*, Rhodocyclales, and Pseudomonadales were isolated previously from the same study site [28,29,30,32,33].

### 3.5. Comparative Analysis of Single Genomes and MAGs Identifies the Cultivated Proxies for Community Analysis

We calculated average nucleotide identity (ANI) values for key phylogenetic groups of MAGs with respect to the cultivated representatives. Among the *Methylococcaceae*, we compared the eight highest-quality MAGs with isolate genomes, all originating from the study site. It is worth noting that most *Methylococcaceae* MAGs were very fragmented (32–447 contigs), even when fulfilling the criteria for medium to high quality. Our hypothesis is that this pattern is due to the presence of multiple closely related strains in the same microcosm, which co-assemble into common scaffolds/genomic bins. Four of the MAGs closely clustered with *Methylobacter* species and one with *Methylosarcina* species, revealing high ANI between the MAGs and the isolates (> 85%; Figure S3). Three of the MAGs represented other *Methylococcaceae* and were clustered with each other while revealing modest (70 ± 2%) ANI with other genomes. None clustered with *Methylomonas* species, in accordance with low abundance of reads classified as *Methylomonas* (not shown) and in agreement with previously published data [15,16]. 

Among the *Methylophilaceae* high-quality MAGs (a total of 24), most clustered closely with the known species of *Methylophilaceae* (a total of 11 genomes were included in the analysis), with ANI values as high as 88 ± 6% (Figure S4). The *Methylophilus* MAGs (a total of 16) were most well assembled, with as few as 1 contigs, suggesting that *Methylophilus* strains tend to ‘bloom’ as low-heterogeneity populations. The *Methylotenera* high-quality MAGs included in the analysis (a total of 8) were more diverse, and these separated into four major phylogenetic groups, all represented by the cultivated isolates (Figure S4). The *Methylotenera* MAGs were less well assembled (10 to 188 contigs) compared to the *Methylophilus* MAGs, suggesting higher heterogeneity for the *Methylotenera* species.

**Figure 3:**
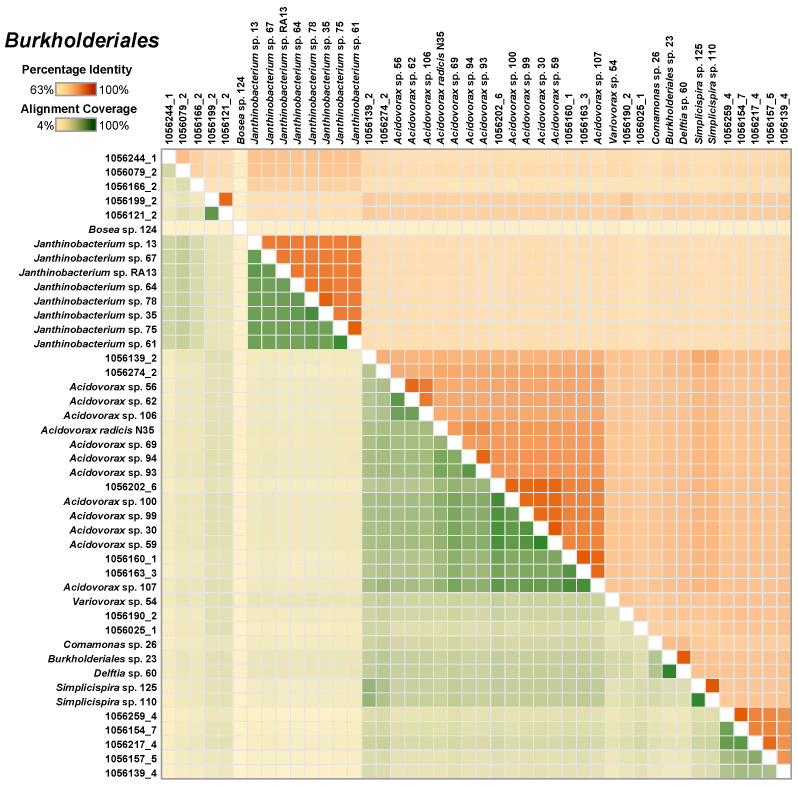
A heatmap depicting the similarity among the select Burkholderiales metaganome assembled genomes (MAGs) and the single-species genomes. The upper right half of the matrix depicts the average nucleotide identity (ANI) between pairs of genomes and the bottom left half depicts alignment coverage between pairs of genomes.

The *Burkholderiales* MAGs (a total of 26) formed five major clusters, with the most represented cluster being *Acidovorax*. These were closely related to the cultivated *Acidovorax* genomes (75 ± 9%; Figure 3). Whereas the *Janthinobacterium* species were easily isolated from methane-fed microcosms, none were represented by MAGs. 

**Figure 4:**
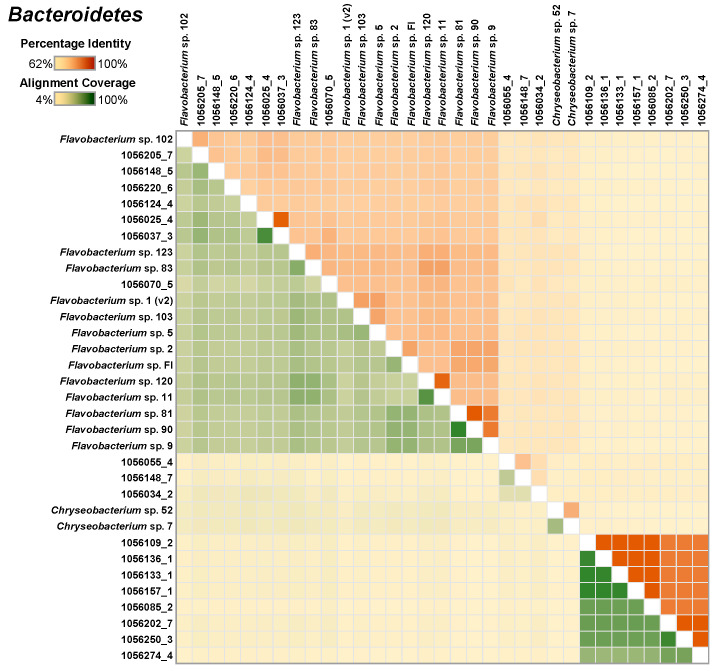
A heatmap depicting the similarity among the select Bacteroidetes MAGs and the single-species genomes. The upper right half of the matrix depicts the average nucleotide identity (ANI) between pairs of genomes and the bottom left half depicts alignment coverage between pairs of genomes.

Similar analysis of 18 Bacteroidetes MAGs, along with 15 isolate genomes, indicated that the newly and the previously isolated species genomes were closely related to the MAGs (Figure 4).

Of the five *Lacunisphaera* MAGs included, four were closely related to each other, while the fifth was more distantly related, and all were only distantly related to the most closely related isolate genome, of *Lacunisphaera limnophila* (Figure S5) [34].

Of a total of eight *Bacteriovorax* MAGs, seven were closely related to each other, while one clustered separately. None were closely related to the top hit known isolates (*Bacteriovorax stolpii*; Figure S6).

### 3.6. Evidence for Simultaneous Expression of Genes for Alternative Methanol Dehydrogenases in Both Methylococcaceae and Methylophilaceae

**Figure 5:**
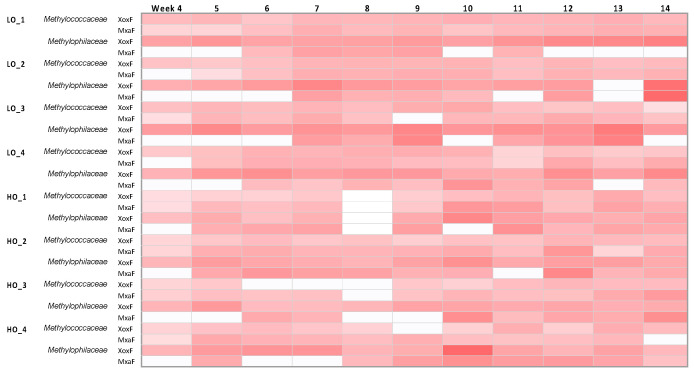
A heatmap depicting expression of *xoxF* and *mxaF* (log2 of transcripts per million, TPM), normalized by total TPM of the respective family (*Methylococcaceae/Methylophilaceae*). Note that reads matching the multiple *xoxF* genes in the *Methylophilaceae* were summed. The HO8, R1 data were omitted due to poor quality.

As expected from prior analyses [17], genes for all the methylotrophy functions were highly expressed in both *Methylococcaceae* and *Methylophilaceae*. It was of special interest to test how genes for the alternative methanol dehydrogenases, XoxF and MxaF, were expressed in the complex microcosms, as it is still not completely understood whether their roles are redundant or complementary. Meantime, it has been already well documented that multiple environmental factors, such as dioxygen level, nitrogen source, and communal life, besides the lanthanides that are the cofactors of XoxF, are involved in the ratio of their respective expression levels [8]. Since we had a lot of complexity in our system, we simplified it by matching the transcripts to the proxy genes from the cultivated genomes (Table S1) and, in the case of *Methylophilaceae* that encoded multiple XoxF enzymes, by combining transcripts matched to all the genotypes of *xoxF* (Table S1). Note that some of the *Methylotenera* ecotypes possess only *xoxF* genes and no *mxaF* [29,30]. This analysis revealed that both *Methylococcaceae* and *Methylophilaceae* expressed both *xoxF* and *mxaF,* in most samples, and the ratios of *xoxF/mxaF* transcripts did not appear to follow any clear pattern (Figure 5, Table S1). In the LO samples, *xoxF* was somewhat higher expressed, and in the HO samples, *mxaF* was somewhat higher expressed, as noted previously for synthetic methanotroph communities [17,19]. However, the ratios were highly variable among the replicates. After the perturbance, variable response was seen among the replicates. These data suggest that, in addition to the regulatory factors previously identified through manipulation of pure cultures and synthetic communities [8], the ratio of XoxF/MxaF may be controlled by additional, yet undiscovered factors. So far, this is the largest dataset reported that demonstrates simultaneous expression of *xoxF* and *mxaF,* in multiple species, over time. It is worth noting that lanthanides were not added to the incubations, as per experimental design.

### 3.7. Hypoxia Stress Response Suggests Competition for Dioxygen

We recently described a unique hypoxia stress response system in *Methylobacter* that involves nitric oxide reductase (NOR), as well as a quorum sensing system regulating the synthesis of a so-far unique secondary metabolite tundrenone [12]. The expression pattern for this system was of special interest to us, especially since the respective genes, *hcp/hcr*, were some of the most highly expressed genes in the microcosms, especially by the *Methylobacter* species (not shown), as noted previously for the hypoxic cultures [12]. Also, as noted previously for the hypoxic cultures [12], the expression of *hcp/hcr* had little correlation with the expression of the (partial) denitrification pathway in *Methylobacter*, with nitrate reductase and other denitrification genes being expressed at less than one order of magnitude compared to *hcp/hcr* (data for *hcp*, annotated as nitric oxide reductase, and *narG* expression are presented in Table S1). Interestingly, the *hcp/hcr* pair was also expressed in the *Methylosarcina* species (Table S1), suggesting that the hypoxia stress response among the species of these two genera must overlap. However, *Methylosarcina* species do not encode respiratory nitrate reductase [28], further suggesting that the major function of NOR is in stress response rather than as part of the denitrification pathway. *Methylosarcina* species also do not encode the additional elements in the stress response that *Methylobacter* species encode, i.e., a quorum-sensing system along with the secondary metabolite tundrenone. Under the conditions used in this study, this system was expressed at a low level in the *Methylobacter* species in most samples, with some exceptions (see Table S1 for *mbaI* expression data). This supports the previous observation on NOR being the main hypoxia response pathway [12]. As the NOR genes were highly expressed not only under the LO but also under the HO condition in this study, this suggests that dioxygen is the main electron acceptor not only for the methantrophs but also for other community members, and that there must be a strong competition for dioxygen within the community. 

As to the terminal cytochrome oxidases, the expression was variable between the ‘traditional’ cytochrome c oxidase and the high-affinity cbb3-type cytochrome oxidase, highlighting the variability in dioxygen level conditions/gene expression among the microcosms that follow individual community trajectories (not shown; these data are available via the IMG/M website).

### 3.8. Metatranscriptome Analysis Uncovers Highly Transcribed Pathways in Burkholderiales and Bacteroidetes, Likely Pinpointing the Mechanisms for the Interspecies Carbon Transfer

The metatranscriptomic reads were matched to the proxy genomes of *Methylococcaceae, Methylophilaceae, Acidovorax*, and *Flavobacterium* strains using both the IMG/M pipeline [23], as well as the custom workflow as described in Materials and Methods. From matching to the *Acidovorax* MAGs, as well as the proxy single genomes assembled as part of this project, we determined that, in sample HO 14, R3, containing the largest number of *Acidovorax* transcripts, some of the most expressed genes detected belonged to the complete respiratory denitrification pathway. The second most expressed pathway was the glyoxylate shunt, whose key enzyme is isocitrate lyase, Icl) [35], and the overlapping tricarboxylic acid (TCA) cycle (Table 1). These data strongly suggest that the main metabolite consumed by *Acidovorax* must be acetate, via the action of acetyl-CoA synthase, whose gene was also highly expressed. Another highly expressed gene encoded acetolactate synthase, and this gene has been previously connected to nitrosative stress response [36]. Note that sample HO 14, R3 is a hypoxic sample (Figure 1). Thus, expression of the denitrification genes appears to be triggered by hypoxia. In comparison, expression of the denitrification genes in an oxic sample, HO 10, R1 was low (Table S5), suggesting that *Acidovorax* switch electron acceptors dependent on dioxygen availability. Notably, genes for the high-affinity cbb3-type cytochrome *c* oxidase were expressed by *Acidovorax* under both LO and HO conditions, but their expression was somewhat higher under the HO condition (Table S5), suggesting that, while oxygen limitation may occur under either condition, nitrate was gradually replacing dioxygen as the preferred electron acceptor by this species, at the onset of hypoxia. All the genes for these highly expressed pathways could be easily identified in all the *Acidovorax* genomes from the cultivated Lake Washington strains, and in the genomes of most of the Burkholderiales belonging to other genera (not shown), suggesting that Burkholderiales other that *Acidovorax* must employ similar metabolic strategies. Interestingly, the key gene of the glyoxylate shunt, *icl,* was missing from the *Janthinobacterium* genomes, potentially suggesting an explanation for their low abundance in the microcosms, due to the lack of the glyoxylate shunt that allows growth on acetate [35]. Thus, the highly expressed genes appear to be parts of the core genome of the Burkholderiales persisting in the microcosms and suggesting that the commonality of their metabolism is related to their function in the methane-oxidizing communities.

Some of the most highly expressed genes encoding the metabolic functions in the *Flavobacterium* species included genes potentially involved in biopolymer transport (Table 2), specifically genes encoding a TonB-dependent transport system involving TonB, ExbD, and ExbB proteins [37] and biopolymer degradation systems of the Sus type [38], potentially suggesting that the Bacteroidetes sustained themselves on scavenging biopolymers produced, likely, by the *Methylococcaceae* and *Methylophilaceae,* and potentially by Burkholderiales. Accordingly, all the genes for sugar metabolism and the TCA cycle [39] were expressed, but not the glyoxylate shunt, suggesting that sugars, rather than acetate, were the main substrates for the Bacteroidetes. Genes for gliding motility were also highly expressed. The genes for all the respective pathways were conserved in all of the genomes of the cultivated *Flavobacterium* species (not shown).

### 3.9. Insights into Metabolisms of Novel Community Members Represented by MAGs

Through MAG assembly, we uncovered, in this study, some novel persistent taxa, even if they were not omnipresent in all of the methane-oxidizing microcosms analyzed. One of these is *Lacunisphaera*, a member of the Verrucomicrobia phylum. The genus *Lacunisphaera* has been recently registered with *Lacunisphaera limnophila* as one of the type strains [34]. The MAGs we assembled in this study were most related to this species but were distant enough to represent two additional novel species of this genus, based on ANI values (see above). *Lacunisphaera* is a representative of Subdivision 4 of the Verrucomicrobia phylum, which is quite distantly related to the division representing verrucomicrobial methanotrophs within this phylum [34]. Whereas, ironically, a gene has been annotated in Bin 129 as *pmoA* (that encodes a subunit of methane monooxygenase in methanotrophs), this gene does not seem to encode a true PmoA (i.e., the protein encoded did not show similarity to the PmoA proteins of the known verrucomicrobial methantrophs, and the gene was not a part of the *pmoCAB* cluster). Other methanotrophy functions were, likewise, missing from the *Lacunisphaera* MAGs we assembled, while all the MAGs encoded most of the genes involved in peptidoglycan biosynthesis (not shown), in agreement with the analysis presented for *L. limnophila* [34]. The cultivated species of *Lacunisphaera* are very versatile in terms of growth substrates [34], and our data agree with the versatile metabolic capabilities of the verrucomicrobial members of the methanotrophic communities characterized in this study. The MAGs we assembled encoded metabolic pathways for utilization of a variety of sugars, as reported by experimental testing [34], and metabolism of polymers such as starch. For the lack of sufficient transcriptomic coverage, we can only hypothesize that *Lacunisphaera* species in the methane-fed microcosms utilized polymers or polymer-derived sugars.

The *Bacteriovorax* species identified in this study represent a new species of this genus. They are most related to *Bacteriovorax stolpii* species [40] but are only distantly related. Whereas they encode the core functions of the predatory *Bacteriovoracaceae* (motility, virulence, Type IV pili) [31], at least one-third of their genomes are novel and without homologs in the *B. stolpii* species. Among these, there are multiple copies of glycosyltransferases and an extensive arsenal of genes for denitrification, along with hypothetical genes (not shown). Unfortunately, the populations of *Bacteriovorax* species were too low for meaningful analysis of the transcripts. Thus, it remains to be determined whether these novel genes contribute to the metabolisms relevant to community living. As to the abundance of the novel glycosyltransferases, it is tempting to speculate that, when in the free-living state, these novel *Bacteriovorax* species feed on biopolymers and potentially gain energy for this metabolism via denitrification.

The Polyangiales MAGs were not closely related to the genomes of any cultivated Polyangiales but were closely related to a MAG assembled from a groundwater metagenome (NCBI Bioproject PRJNA514088). Although only distantly related to the known Polyangiales predatory species, the respective MAGs encoded three types of secretion systems, T3SS, T4SS, and T6SS, known to be involved in predation [41]. They also encoded chitinase, multiple hydrolases, and peptidases, suggesting a predatory lifestyle.

The Thiotrichales MAGs present a very puzzling case. Whereas they represented organisms only distantly related to the parasitic Thiotrichales of the genus *Francisella* (<87% 16S rRNA gene identity), they appeared to have small genomes (~1.8 Mb), typical of the parasitic species. Whereas no genes for Type VI secretion system implicated in pathogenicity of *Francisella* [42] were present in the genomes, they encoded multiple ankyrin repeat homology domain-containing proteins (Anks; 31 gene copies in each MAG), along with the genes for Type IV secretion system, implicated in pathogenesis in other species [43]. As we have not recovered many eucaryotic sequences in either methagenome, it is tempting to speculate that these organisms may have been free-living in the microcosms. Both genomes encoded sugar metabolism, acetate metabolism, and the tricarboxylic acid (TCA) cycle, as well as other traditional energy generation pathways, such as ATPase and respiratory complex I.

## 4. Discussion

**Figure 6:**
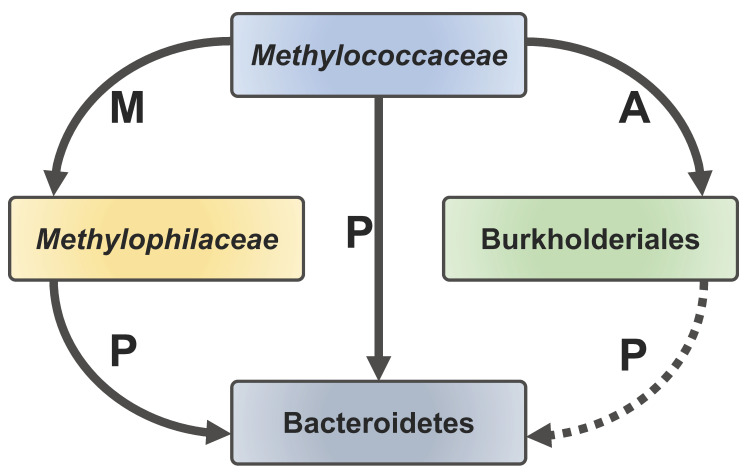
A schematic of carbon transfer among the core functional guilds involved in methane consumption. *Methylococcaceae* excrete methanol (M) and acetate (A)/other organics that are consumed, respectively, by *Methylophilaceae* and certain Burkholderiales. *Methylococcaceae, Methylophilaceae*, and potentially Burkholderiales excrete polymeric substances (P) that are consumed by Bacteroidetes.

In this study, our aim was to obtain a high-resolution insight into the social life of the methanotrophs, which we previously observed to form communities with other, nonmethanotrophic organisms. The data we present here support our observations from earlier experiments that applied lower-resolution sequencing approaches to the natural complex communities [15,16], as well as observations from manipulations of very simple or more complex synthetic communities [17,18]. We observed, yet again, that the *Methylococcaceae* and the *Methylophilaceae* dominated the methanotrophic communities, while we also obtained solid evidence for the additional functional guilds persisting in these communities, especially the Burkholderiales and the Bacteroidetes. We showed that Burkholderiales are equipped with the capability of utilizing the glyoxylate shunt, enabling them to utilize acetate and potentially other products of fermentative metabolism by the *Methylococcaceae*, while Bacteroidetes likely utilize a variety of polymeric substances released by *Methylococcaceae, Methylophilaceae*, and, potentially, Burkholderiales. With these natural communities, we observed a lot of variation in terms of the replicates, highlighting the problems with investigating complex natural communities. Interestingly, we also observed wide variation in expression of some of the major genes in methylotrophy. For example, the ratio of expression of genes encoding the alternative methanol dehydrogenases (XoxF versus MxaF) that are key enzymes in both the *Methylococcaceae* and the *Methylophilaceae* was highly variable, further pinpointing the complexity of their regulation. Whereas we previously speculated about the nature of the metabolic connections of the Burkholderiales and the Flavobacteriales, as parts of the communities, we now present some compelling data from the transcriptomics as to the specifics of the respective metabolisms: The Burkholderiales appear to consume acetate, which would be a fermentation product of the hypoxic metabolism of *Methylococcaceae* [44], while also engaging in denitrification as an alternative energy metabolism. The Bacteroidetes appear to be opportunistic consumers of a variety of biopolymers produced by the primary methane-consuming community members (Figure 6). Additionally, predatory species appeared to be involved, specifically the novel *Bacteriovorax* species. Our data are not conclusive as to which species serve as the prey for the novel *Bacteriovorax*. These could be *Methylococcaceae* or *Methylophilaceae*, but we could not find a solid correlation between the populations of the respective species. In the future, experiments could be designed to determine the preferred prey(s) for these novel *Bacteriovorax* species. The presence of the novel Opitutae species (*Lacunisphaera*), which are nonmethanotrophs, in the methanotrophic communities is also intriguing, and these may be generalist species feasting on the polymers or sugars released by other species. Overall, our present study further suggests that the aerobic methanotrophs, whether acting under oxic or hypoxic conditions, tend to support diverse communities, apparently feeding off the methane-derived organics. These communities are not random but are predetermined by specific metabolic capabilities. It also appears that the harmony of such communities could be affected by the predatory species. Overall, we conclude that, while the natural community manipulations provide novel and important insights into the function of the methane-consuming communities, a balance between natural versus synthetic community experimental designs holds potential for deciphering the complicated relationships between the true methanotrophs and their community partners.

## Figures and Tables

**Table 1:** Fifty highly expressed *Acidovorax* genes in sample HO 14, R3.

Normalized Reads	Protein Annotation
4406.72	nitrous-oxide reductase
4361.95	nitric oxide reductase, NorB subunit
4017.11	dissimilatory nitrite reductase (NO-forming)
2591.06	cytochrome c oxidase cbb3-type subunit 1
2058.85	nitric oxide reductase, NorC
1840.81	L-glutamine synthetase
1577.16	nitrate reductase alpha subunit
1514.15	cytochrome c oxidase cbb3-type subunit 2
1511.56	isocitrate lyase
1464.03	respiratory nitrate reductase beta subunit
941.95	nitrogen regulatory protein P-II family
723.80	nitrate/nitrite transporter NarK
704.82	Aconitase
685.12	malate dehydrogenase (NAD)
667.33	nitrate reductase gamma subunit
642.85	nitric oxide reductase NorQ
621.24	type IV pilus assembly protein PilA
617.12	succinyl-CoA synthetase alpha subunit
615.04	succinyl-CoA synthetase beta subunit
588.58	succinate dehydrogenase subunit B
553.90	acetolactate synthase, small subunit
518.04	acetolactate synthase, large subunit
517.77	isocitrate dehydrogenase
425.76	cytochrome c oxidase cbb3-type subunit 3
424.15	2-isopropylmalate synthase
420.08	citrate synthase
400.64	acetyl-coenzyme A synthetase
364.95	succinate dehydrogenase subunit A
309.10	isocitrate dehydrogenase
289.19	dihydrolipoamide dehydrogenase
288.81	isocitrate lyase
283.16	glyceraldehyde-3-phosphate dehydrogenase
222.93	succinate dehydrogenase subunit C
217.08	Enolase
214.48	glutamate synthase (NADH) large subunit
198.58	6-phosphogluconate dehydratase
196.19	respiratory nitrate reductase chaperone NarJ
194.83	pyruvate dehydrogenase E1 component
167.72	2-oxoglutarate dehydrogenase E1 component
167.20	phosphoenolpyruvate synthase
160.71	NosR/NirI nitrous oxide reductase regulator
160.36	Transketolase
145.43	acetyl-CoA carboxylase carboxyltransferase alpha
123.76	pyruvate dehydrogenase E2 component
116.03	propionyl-CoA synthetase
114.66	glutamate synthase (NADH) small subunit
111.75	fructose-bisphosphate aldolase
110.33	succinate dehydrogenase subunit D
107.42	2-oxoglutarate dehydrogenase E2 component
105.95	malate synthase

**Table 2:** Fifty highly expressed *Flavobacterium* genes in sample HO 10, R1.

Normalized Reads	Protein Annotation
8811.54	preprotein translocase subunit SecE
2575.34	protein TonB
2574.02	outer membrane transport energization protein ExbB
2170.64	glyceraldehyde 3-phosphate dehydrogenase
1962.49	preprotein translocase subunit SecG
1556.71	biopolymer transport protein ExbD
1468.84	enolase
1430.38	gliding motility associated protien GldN
1359.97	pyruvate dehydrogenase E1 component beta subunit
1303.32	protein involved in gliding motility GldL
1205.79	fructose-bisphosphate aldolase
1179.97	citrate synthase
1137.43	phosphoenolpyruvate carboxykinase
1072.83	gliding motility-associated lipoprotein GldJ
1058.14	outer membrane transport energization protein ExbD
1053.74	protein involved in gliding motility GldK
1028.16	acetyl-CoA carboxylase carboxyl transferase beta
956.30	glycine cleavage system H protein
849.13	cytochrome c oxidase cbb3-type subunit I/II
840.36	malate dehydrogenase
807.56	triosephosphate isomerase
717.63	glycine hydroxymethyltransferase
708.77	dihydrolipoamide dehydrogenase
682.67	pyruvate dehydrogenase E1 component alpha subunit
681.09	succinate dehydrogenase iron-sulfur subunit
673.95	succinyl-CoA synthetase beta subunit
660.97	transaldolase
625.88	glutamine-fructose-6-phosphate transaminase
580.02	acetyl-CoA carboxylase biotin carboxyl carrier protein
563.70	acetyl-coenzyme A synthetase
535.31	starch-binding associating with outer membrane
531.84	succinyl-CoA synthetase alpha subunit
525.95	acetyl-CoA carboxylase carboxyl transferase alpha
514.91	aconitase
509.61	arabinose-5-phosphate isomerase
508.92	TonB-linked outer membrane protein, SusC/RagA
507.51	phosphoglycerate kinase
473.35	2-oxoglutarate dehydrogenase E2 component
472.65	pyruvate dehydrogenase E2 component
439.43	acetyl-CoA carboxylase, biotin carboxylase subunit
437.56	6-phosphofructokinase
427.50	ribose-phosphate pyrophosphokinase
394.14	biopolymer transport protein ExbD
377.35	transketolase
370.57	starch synthase
363.35	transketolase
360.88	2-oxoglutarate dehydrogenase E1 component
312.13	2-oxoglutarate dehydrogenase E2 component
190.55	Aconitase
190.11	triosephosphate isomerase

## Supplementary Materials

The following are available online at https://www.mdpi.com/2076-2607/8/10/1614/s1, Figure S1. PCA analysis of core taxa abundances (LO versus HO), over 11 sampling points. Figure S2. Transcriptomics-based community dynamics in microcosms transitioning from ‘low’ to ‘high’ (a) and from ‘high’ to ‘low’ (b) oxygen conditions, at the family/order level (left) and at the genus level (right). The switch between the conditions at week 10 is marked by an arrow. R1-R4, replicates. The HO8, R1 data were omitted due to poor quality. Figure S3. A heatmap depicting the similarity among the select *Methylococcaceae* MAGs and the single species genomes. The upper right half of the matrix depicts the average nucleotide identity (ANI) between pairs of genomes and the bottom left half depicts alignment coverage between pairs of genomes. Figure S4. A heatmap depicting the similarity among the select *Methylophilaceae* MAGs and the single species genomes. The upper right half of the matrix depicts the average nucleotide identity (ANI) between pairs of genomes and the bottom left half depicts alignment coverage between pairs of genomes. Figure S5. A heatmap depicting the similarity among the select *Lacunisphaera* MAGs and the closest relative genome. The upper right half of the matrix depicts the average nucleotide identity (ANI) between pairs of genomes and the bottom left half depicts alignment coverage between pairs of genomes. Figure S6. A heatmap depicting the similarity among the select *Bacteiovorax* MAGs and the closest relative genomes. The upper right half of the matrix depicts the average nucleotide identity (ANI) between pairs of genomes and the bottom left half depicts alignment coverage between pairs of genomes. Table S1. Select gene transcript counts. Table S2. Genome statistics for the Burkholderiales and Bacteroidetes cultivated in this study. Table S3. Original MAG assembly statistics. Table S4. Statistics for high and medium quality MAGs generated in this study. Table S5. Select highly expressed genes in *Acidovorax*.
