# Supplementary material for: Metagenomic Insight into Environmentally Challenged Methane-Fed Microbial Communities
Source: Microorganisms. 2020 Oct 20;8(10):1614. doi: 10.3390/microorganisms8101614 (PMC7589939; doi:10.3390/microorganisms8101614)
Supplement: Supplementary file 1 [file microorganisms-08-01614-s001.zip › Figure S5.pdf]

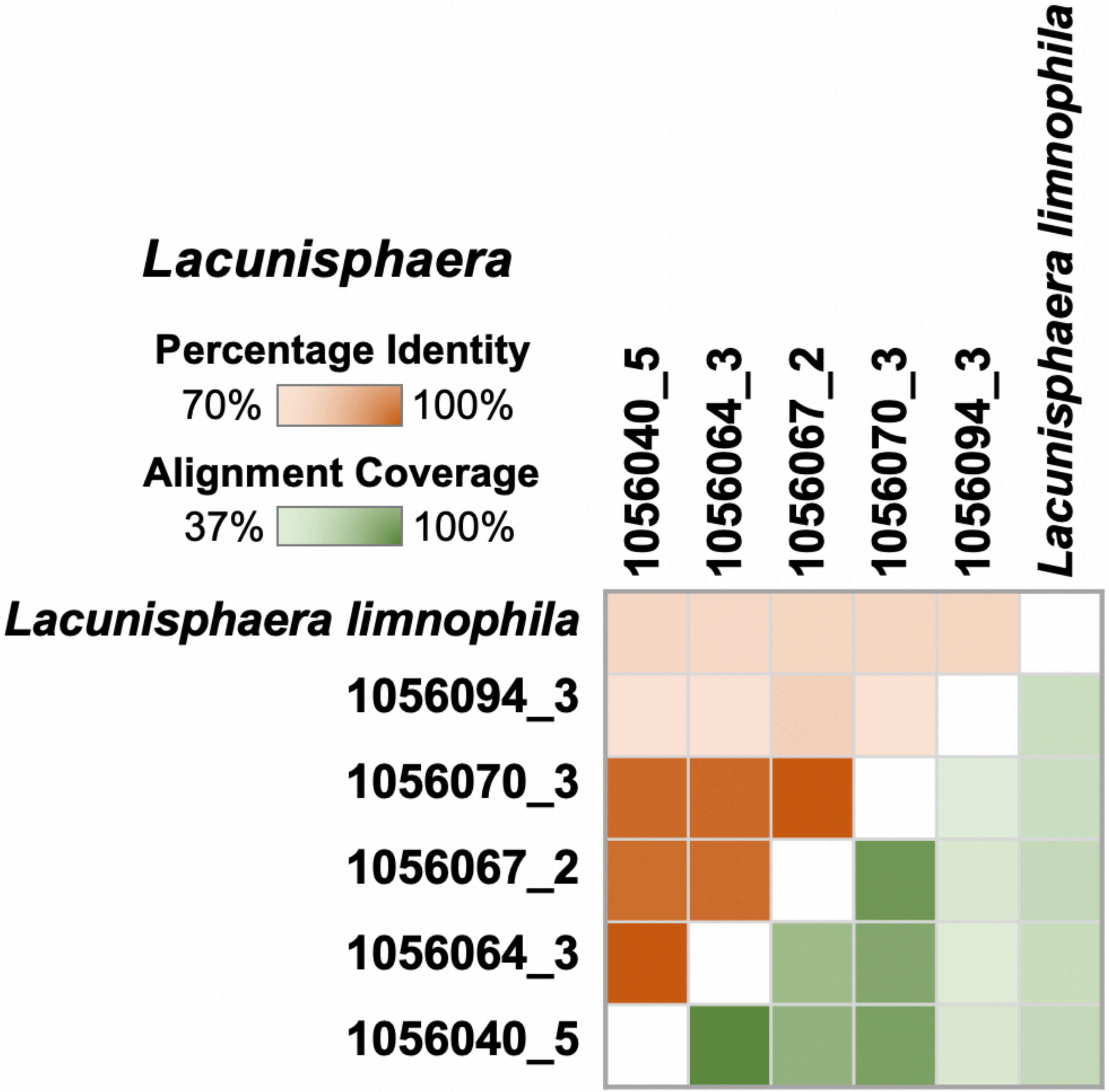

Lacunisphaera
Percentage Identity
70%
100%
Alignment Coverage
37%
100%
1056040_5
1056064_3
1056067_2
1056070_3
1056094_3
Lacunisphaera limnophila
Lacunisphaera limnophila
1056094_3
1056070_3
1056067_2
1056064_3
1056040_5
