# Supplementary material for: Metagenomic Insight into Environmentally Challenged Methane-Fed Microbial Communities
Source: Microorganisms. 2020 Oct 20;8(10):1614. doi: 10.3390/microorganisms8101614 (PMC7589939; doi:10.3390/microorganisms8101614)
Supplement: Supplementary file 1 [file microorganisms-08-01614-s001.zip › Figure S6.pdf]

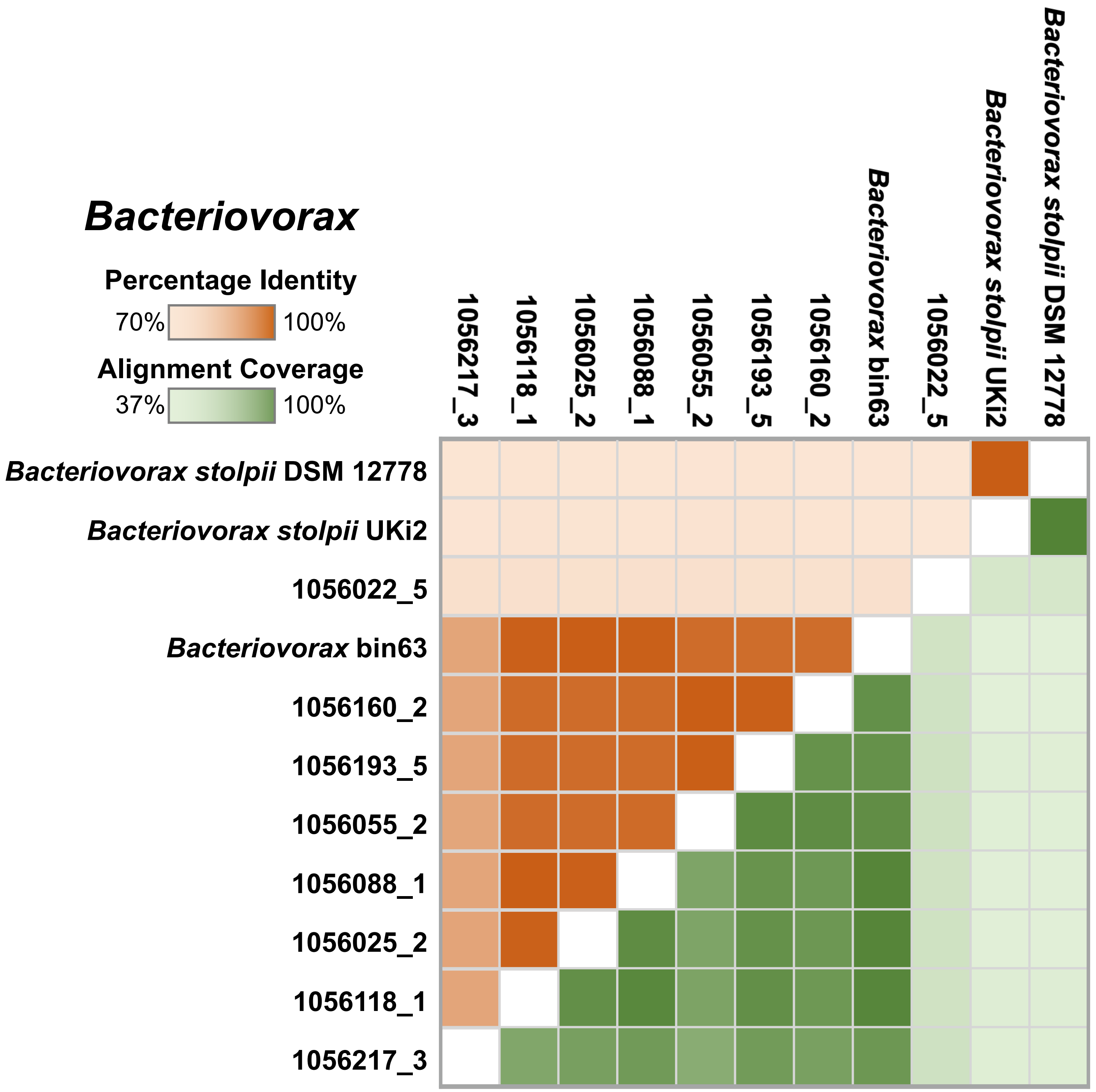
Bacteriovorax
Percentage Identity
70%
100%
Alignment Coverage
37%
100%
1056217_3
1056118_1
1056025_2
1056088_1
1056055_2
1056193_5
1056160_2
Bacteriovorax bin63
1056022_5
Bacteriovorax stolpii UKi2
Bacteriovorax stolpii DSM 12778
