# Supplementary figures and images for: Metagenomic Insight into Environmentally Challenged Methane-Fed Microbial Communities
Source: Microorganisms. 2020 Oct 20;8(10):1614. doi: 10.3390/microorganisms8101614 (PMC7589939; doi:10.3390/microorganisms8101614)

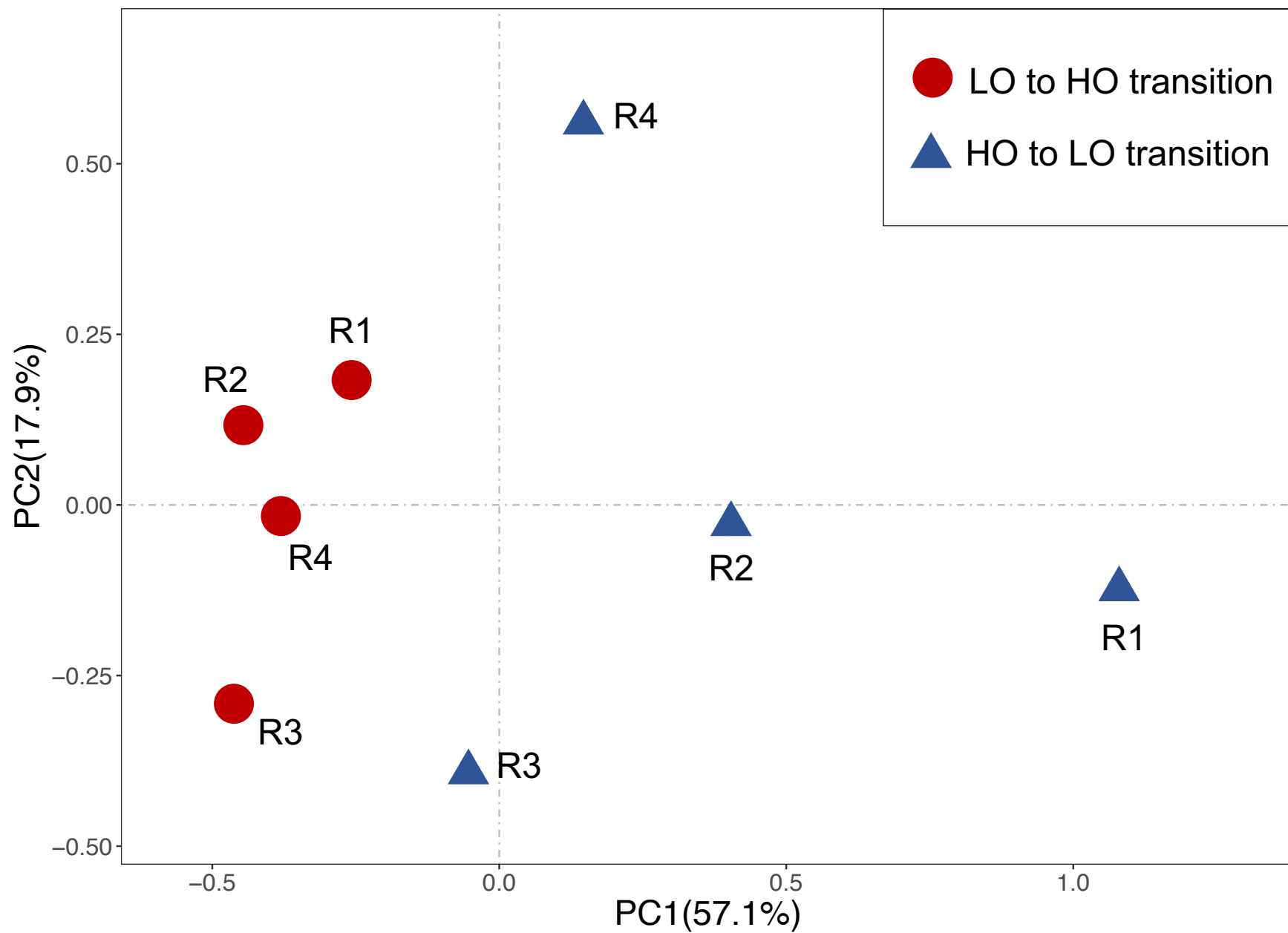

Supplement: Supplementary file 1 [file microorganisms-08-01614-s001.zip › Figure S1.pdf]

**R 1**

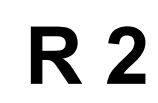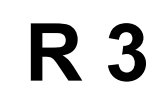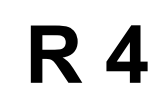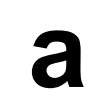

## R 1

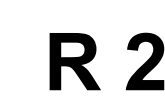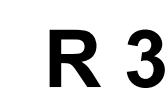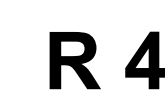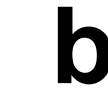

Supplement: Supplementary file 1 [file microorganisms-08-01614-s001.zip › Figure S2.pdf]

**Percentage Identity**  
68% 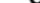 100%

**Alignment Coverage**  
13% 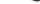 100%

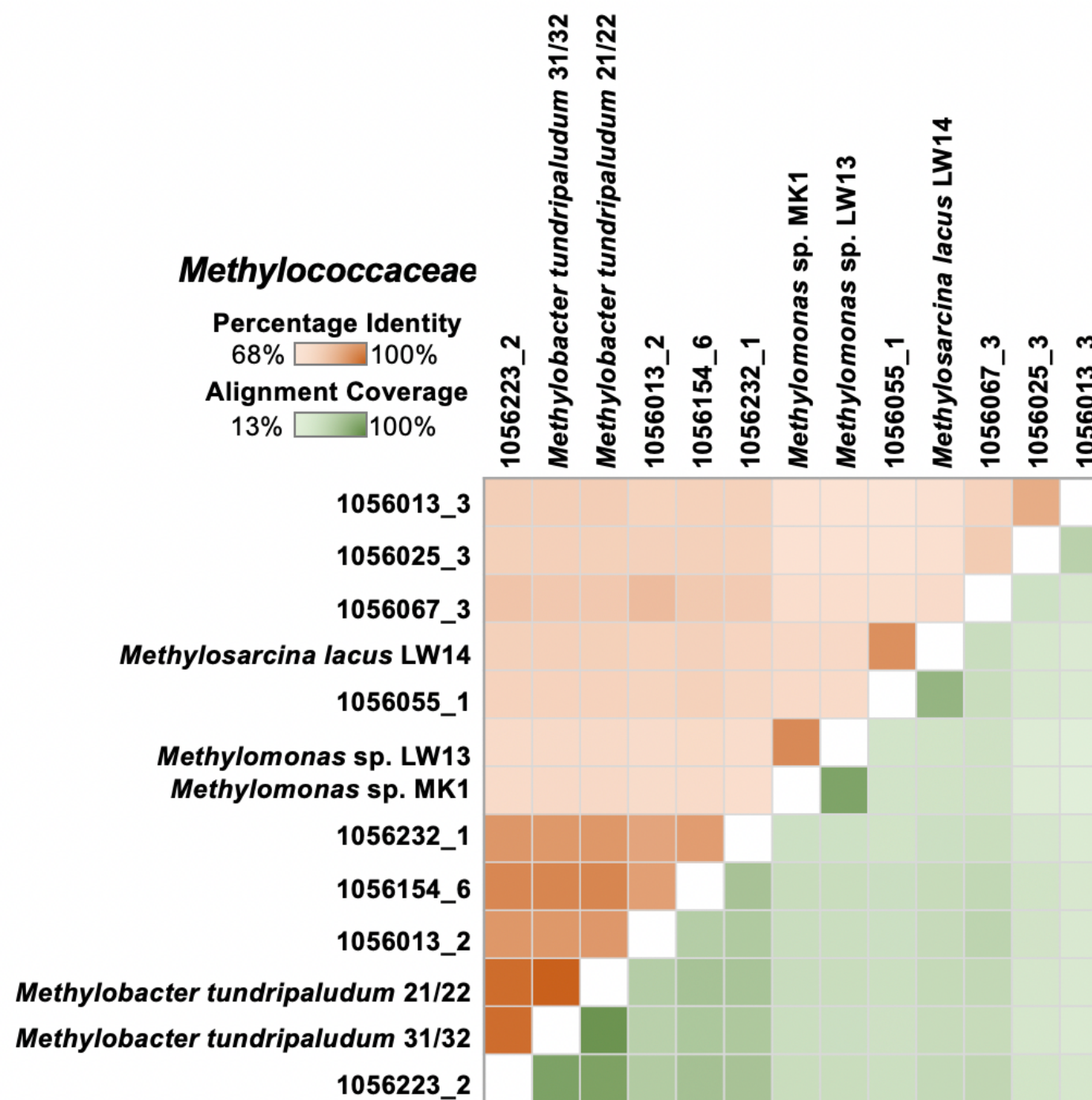

Supplement: Supplementary file 1 [file microorganisms-08-01614-s001.zip › Figure S3.pdf]

**Percentage Identity**  
68% 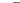 100%

**Alignment Coverage**  
18% 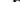 100%

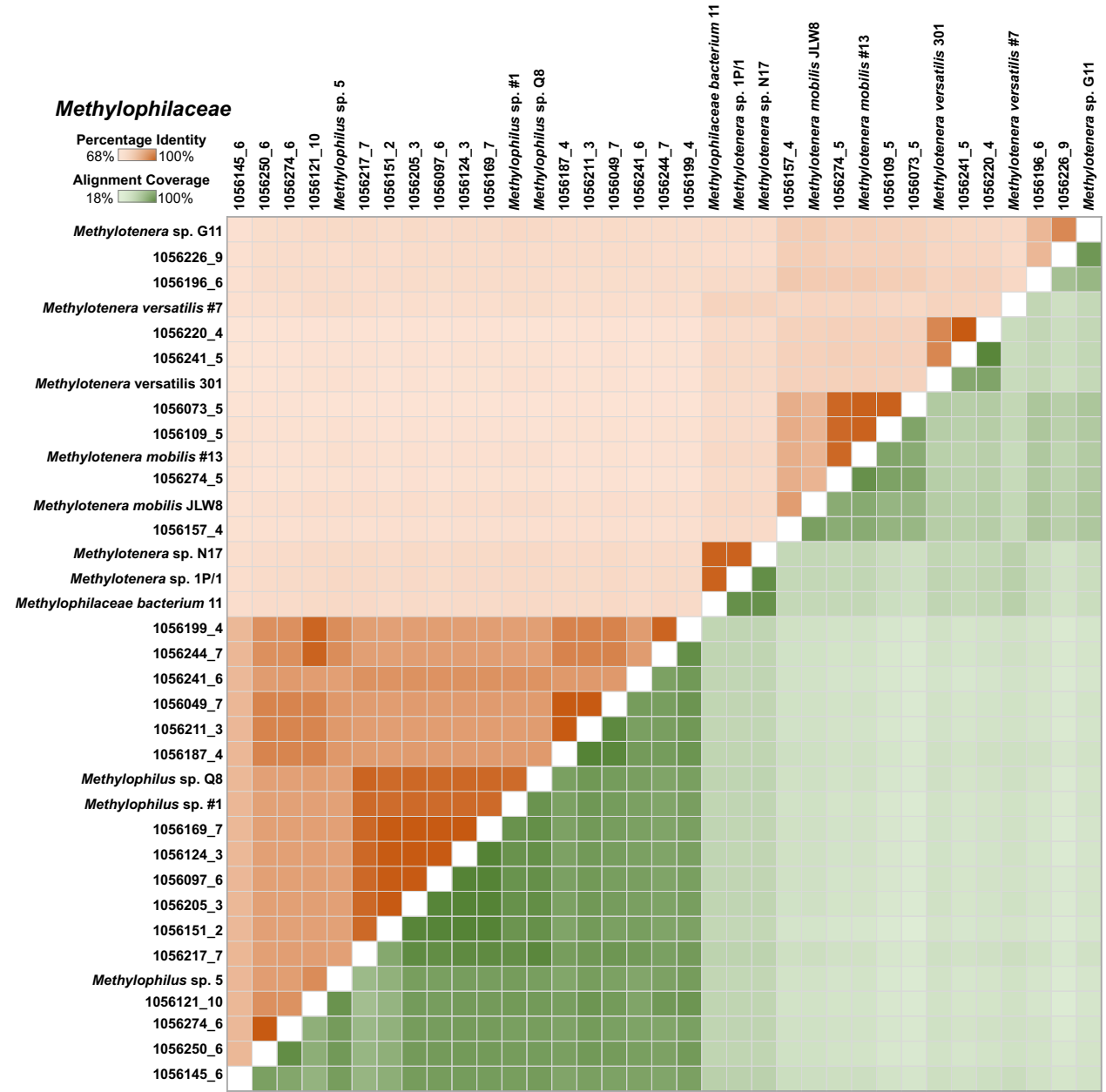

Supplement: Supplementary file 1 [file microorganisms-08-01614-s001.zip › Fugure_S4.pdf]
